# Simultaneous Determination of Vitamins D3 (Calcitriol, Cholecalciferol) and K2 (Menaquinone-4 and Menaquinone-7) in Dietary Supplements by UHPLC

**DOI:** 10.3390/molecules26226982

**Published:** 2021-11-19

**Authors:** Anca Becze, Vanda Liliana Babalau Fuss, Daniela Alexandra Scurtu, Maria Tomoaia-Cotisel, Aurora Mocanu, Oana Cadar

**Affiliations:** 1 Research Institute for Analytical Instrumentation, National Institute of Research and Development for Optoelectronics INOE 2000, 67 Donath Street, 400293 Cluj-Napoca, Romania; anca.becze@icia.ro (A.B.); vanda.fuss@icia.ro (V.L.B.F.); daniela.scurtu@icia.ro (D.A.S.); 2 Faculty of Chemistry and Chemical Engineering, Research Center of Physical Chemistry, Babeş-Bolyai University, 11 Arany Janos Street, 400028 Cluj-Napoca, Romania; mcotisel@gmail.com (M.T.-C.); mocanu.aurora@gmail.com (A.M.)

**Keywords:** UHPLC, method validation, calcitriol, cholecalciferol, menaquinone-4, menaquinone-7

## Abstract

The content and composition of dietary supplements is of great interest due to their increasing consumption and variety of available brand offered in the market. Accurate determination of vitamins is important for the improvement of dietary supplement quality and nutrition assessments. In this regard, the simultaneous determination of vitamin D3 (calcitriol—CT and cholecalciferol—CHL) and K2 (menaquinone-4—MK-4 and menaquinone-7—MK-7) in dietary supplements was developed by using ultra-high-pressure liquid chromatography (UHPLC). The overall runtime per sample was above 35 min, with the retention times of 2.40, 6.59, 7.06, and 32.6 min for vitamin D3 (CT and CHL) and vitamin K2 (MK-4 and MK-7), respectively. The limits of detection and limits of quantification for the target nutritional compounds ranged between 0.04–0.05 µg/mL, respectively. The validation results indicated that the method had reasonable linearity (*R*^2^ ≥ 0.9990), good recovery (>82%), satisfactory intra-day precision (≤1.9%) and inter-day precision (≤3.5%), and high selectivity and specificity. The validated UHPLC method was demonstrated to be precise, accurate, and robust for the simultaneous determination of vitamins D3 (CT and CHL) and K2 (MK-4 and MK-7) in dietary supplements.

## 1. Introduction

Worldwide, the vitamins and minerals are the most widespread dietary supplements used by populations. The provided amount of micronutrients varies from less to much more than the recommended intakes, making them important contributors to total intakes [1]. However, a well-balanced diet is the key for population-based primary prevention of chronic diseases [2].

Vitamin D includes a group of fat-soluble seco-steroids, the most important forms being vitamin D3 (cholecalciferol) and D2 (ergocalciferol). Vitamin D2 is derived from plant sources, while vitamin D3 is a fat-soluble vitamin that is mainly synthesized by the human skin when exposed to sunlight, but can also be ingested by foods such as fatty fish, eggs, and dairy products [3]. Vitamin D, a prohormone, stimulates the optimal intestinal calcium and phosphorous absorption, and promotes the healthy growth and remodeling of bone. The vitamin D deficiency is associated with increased risk of cardiovascular diseases (hypertension, heart failure and ischemic heart disease) and acute respiratory tract diseases (viral infections) [4]. The most active form of vitamin D3 (1,25-dihydroxyvitamin D (1,25(OH)2D3), calcitriol) exists in liver and kidney after hydroxylation [5]. The vitamin D3 supplementation is encouraged in order to restore the calcitriol (CT) concentrations and it is considered to be safe with doses up to 4000 international units (IU) per day. Vitamin D3 supplements are available in 400–2000 IU capsules and liquid drops at pharmacies and supermarkets [3]. Fortified foods and dietary supplements are important sources of vitamin D to meet the requirements, but large doses can cause poisoning. Therefore, the accurate measurement of vitamin D is essential in clinical studies, food industry, and nutritional chemistry [6].

Vitamin K_2_ (menaquinone) is a natural form of vitamin K that occurs in fermented dairy products, while the other form of vitamin K, vitamin K_1_ (phylloquinone), is abundant in leafy green vegetables. Vitamin K_2_ refers to a series of naphthoquinone derivatives and is also called menaquinone-n (MK-n, *n =* 1–14), where n is the number of isoprene units [1]. Vitamin K_2_ exhibits positive impact on the cardiovascular disease, osteoporosis, parathyroid illnesses, and cerebral palsy [7,8]. Some studies suggest that the bioavailability of vitamin K_2_ is related to the length of the side chain, the medium-length menaquinone (MK-7) being more bioavailable than short- (MK-4) or long-chain (MK-8 or MK-9) menaquinones. Of these, MK-7 and MK-7 are the most active forms and have been used as nutrients by the food industry and as nutritional supplements [9]. While the presence of menaquinones in food is well-known and its demonstration of health benefits is increasing [7], to the best of our knowledge, the publications on reliable and accurate quantification methods (and efficient quantitative extraction methods) from dietary supplements are scarce.

Whereas the vitamins D3 and K2 have separately been recognized as beneficial in balancing calcium for bone and cardiovascular health, recent studies suggest that vitamin D improves the concentration of vitamin K-dependent bone protein and persuades the bone formation, concomitantly with the stimulation of osteoblastic gene expression [10]. Due to their promoting activity for bone and cardiovascular health, the vitamins D3 and K2 are incorporated into the multivitamin formulation, along with calcium. Consequently, their determination in dietary supplements is required and urgently needed, but no official, validated method exists for the simultaneous measurement of vitamins D3 and K2.

Various methods have been used for the determination of fat-soluble vitamins in different matrices such as biological fluids, foods, and plant material by, namely spectrophotometry, fluorimetry, electrochemistry, immunoassay, capillary electrophoresis, and chromatography [6,7,11,12]. Of these, the chromatographic methods such as thin-layer chromatography (TLC), gas chromatography (GC), and high-performance liquid chromatography (HPLC) with electrochemical, spectrophotometric, fluorimetric, or mass spectrometric detection methods are suitable and widely used for the determination of vitamins D3 and K2 [6,7,11,12]. When dealing with complex matrices, a comprehensive sample preparation is necessary to eliminate interferences in the HPLC method from other compound having chemical formulas and structures [6].

Instead of good sensitivity of the above-mentioned techniques, their cost and complexity are challenging for the routine quality control laboratories of pharmaceutical industries. The goal of this study was to optimize and validate an ultra-high-pressure liquid chromatographic (UHPLC) method for the simultaneous determination of vitamins D3 (cholecalciferol—CHL and calcitriol—CT) and K2 (MK-4 and MK-7) in pharmaceutical formulations. The method was applied to quantify the vitamins D3 (CHL and CT) and K2 (MK-4 and MK-7) and to evaluate their distribution in commercially available dietary supplements with different composition and concentrations. The paper is important for the quality control analytical laboratories dealing with the determination of vitamin D3 (CT, CHL) and K2 (MK-4, MK-7) in dietary supplements by UHPLC, since it presents a simultaneous and fully-validated method for this purpose.

## 2. Results and Discussion

The validation study for the simultaneous determination of vitamin D3 (CT and CHL) and K2 (MK-4 and MK-7) in dietary supplements was performed to characterize the proposed analytical method in terms of conformity of chromatographic parameters (tailing factor, selectivity factor, resolution, theoretical plates), specificity, working and linear ranges, limit of detection (LoD) and limit of quantification (LoQ), accuracy, precision, and recovery.

### 2.1. Chromatographic Parameters

**Figure 1:**
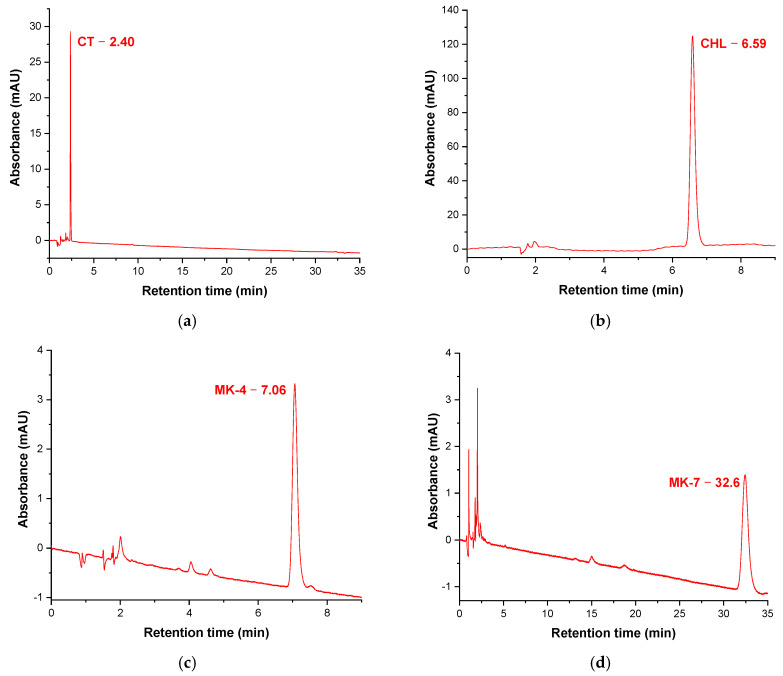
HPLC chromatograms of the analyzed D3 and K2 vitamins: (**a**) calcitriol, CT; (**b**) cholecalciferol, CHL; (**c**) menaquinone-4, MK-4; (**d**) menaquinone-7, MK-7.

The calibration curves of vitamins analyzed (CT, CHL, MK-4, and MK-7) are presented in Figure 1a–d. The total analysis time was 35 min due to the big differences in the structure of D3 and K2 vitamins, especially MK-7, which has a high molecular weight (646 g/mol) compared to that of CT (417 g/mol).

Chromatography Data System software (version 7.3, Thermo Scientific Dionex Chromeleon 7) was used for data acquisition and processing; the chromatographic parameters were calculated according to the European Pharmacopeia [13]. The chromatographic parameters displayed an excellent peak shape, resolution, and high number of theoretical plates (Table 1). The RSD of retention time was lowest for CHL (0.06%) and highest for CT (0.87%). According to Shabir et al., the resolution should be above 2 between the peak of interest and the closest eluting potential interference (impurity, excipient, degradation product, internal standard, etc.), and the theoretical plates should be >2000 [14]. The high values of the theoretical plates (N > 10,500) proved the greater efficiency of the column [14,15]. The asymmetry factors of all investigated compounds were a little above 1 (T ≤ 2 according to the ICH guidelines [14,15]), indicating a small tailing, but precise quantification measurements. The resolution could not be calculated for MK7 because there was no other peak near the retention time (32.6 min).

### 2.2. Linearity, Limit of Detection (LOD), and Limit of Quantification (LOQ)

**Figure 2:**
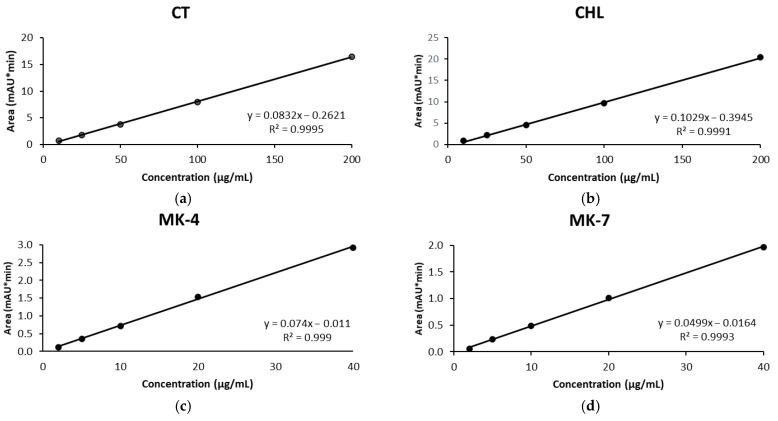
Calibration curves of: (**a**) calcitriol, CT, (**b**) cholecalciferol, CHL, (**c**) menaquinone, MK-4, and (**d**) menaquinone-7, MK-7.

The concentration intervals were chosen based on the average content of vitamins D3 and K2 found in commercial dietary supplements. Mixed standard solutions with different concentrations of four vitamins (CT and CHL—vitamin D3 and MK-4 and MK-7—vitamin K2) were examined by UHPLC-DAD. Five-point calibration curves were performed to evaluate the linearity. The regression coefficient for all calibration curves was greater than 0.999 (Figure 2a–d). The obtained results were better than those found in the literature, which considers that a regression coefficient above 0.99 is satisfactory [15,16,17,18,19,20,21,22,23].

The LOD and LOQ of the target analytes are presented in Table 2. The goal of this study was to reduce the detection and quantification limits, LOD and LOQ, to as low as reasonably achievable, while still attaining good accuracy and precision [14].

The calculated LOQs for each target analyte, 0.04 µg/mL for CT and MK-4 and 0.05 µg/mL for CHL and MK-7, were verified with six sample replicates at LOQ level (Table 3). The lowest RSD value was obtained for CT (13.1%) and the highest for CHL (17.8%). Since the RSD values were lower than 20% and the recovery was in the range of 80–120% compared to the theoretical value (true), the LOQs calculated were accepted as valid [24,25,26]. The proposed method provided recoveries in the range of 104–115% (Table 3), which justified the suitability of UHPLC technique for the intended application, namely simultaneous determination of vitamin D3 (CT and CHL) and K2 (MK-4 and MK-7) in dietary supplements. Furthermore, the main advantages of UHPLC over classical HPLC are ease of operation, high selectivity, and minimal solvent consumption [15].

### 2.3. Recovery, Selectivity and Specificity

Three parallel samples were analyzed by spiking known D3 and K2 concentrations in similar dosage forms (Table 4). The average recovery was found to be 81% for CHL, 85% for CL, 83% for MK-4, and 82% for MK-7, which justified the appropriateness of UHPLC technique for the envisioned applications. The recovery values were lower than those obtained for vitamins D3 and K2 (MK-7) in pharmaceutical solid dosage formulation by Jehangir [15], but higher than those obtained for vitamin K2 (MK-4 and MK-7) in dietary supplements by Bhandari [16]. Since no change in the retention times and no peak from the matrix that eluted near the peaks of interest were remarked, we concluded that the proposed method has high selectivity and specificity.

### 2.4. Precision

The method precision represents the degree of repeatability and is expressed as the percent relative standard deviation (%RSD) [14] The obtained results for the intra assay precision for the concentration of 40.0 µg/g for vitamin D3 (CT and CHL) and 40.0 µg/g for vitamin K2 (MK-4 and MK-7) are presented in Table 5. The %RSD after the repeatability test was under 2% for each analyte, the best %RSD being obtained for CT (1.27%).

For precision (reproducibility) studies, the vitamins were analyzed in three different lots with different concentrations (i.e., 10.0, 12.0, and 30.0 µg/mL for vitamin D3 (CT and CHL), and 5, 6 and 10. µg/mL for vitamin K2 (MK-4 and MK-7)) over a period of five consecutive days (Table 6). RSD values ranged between 2.33–3.57% (lot 1, with the lowest concentrations), 1.98–3.41% (lot 2), and 1.31–3.25% (lot 3, with the highest concentrations). Bhandari reported similar results for the validation method of vitamin K2 (MK-4 and MK-7) for the Association of Official Analytical Chemists (AOAC) [16].

### 2.5. Robustness

The method robustness represents the ability of an analytical method to remain unaffected by the small variations of one or more parameters [16]. In our case, the robustness was evaluated by slight changes in the chromatographic parameters such as column temperature, flow rate, mobile phase ratio, and wavelength. Then, the vitamin contents besides chromatographic parameters such ass retention time, tailing factor, and resolution were determined. The obtained results presented in Table 7 demonstrated that the effects of the deliberate changes in chromatographic conditions were neglectable and that the proposed UHPLC method was robust for its intended applications

### 2.6. Analysis of Commercial Dietary Supplements and Synthetic Drugs in Capsule Form

**Figure 3:**
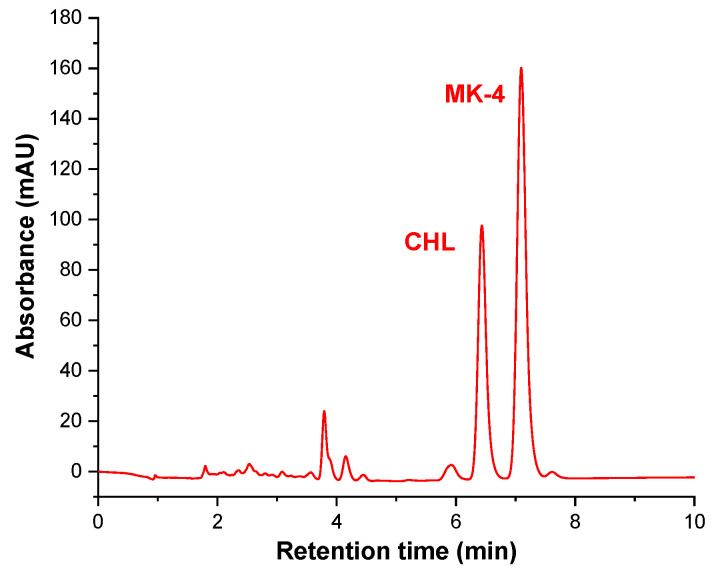
Chromatogram plot of dietary supplement DS-CHL@MK4.

The applicability of the validated UHPLC method was evaluated by examining six commercial dietary supplements and two commercial synthetic drugs (Table 8) with different reported concentrations of vitamins D3 (CT and CHL) and K2 (MK-4 and MK-7). The removal of excipients with an extraction step before the UHPLC analysis was considered unnecessary. The chromatogram of the dietary supplement A containing CHL and MK-4 is presented in Figure 3.

The results obtained for the analysis of six dietary supplements and two synthetic drugs are shown in Table 8; the results are expressed as the average of six measurements. The recovery and RSD values ranging between 96–103% (D3) and 100–105% (K2), and 1.38–4.42% (D3) and 1.21–1.90% (K2), respectively, indicated that the validated UHPLC method is sufficiently precise and accurate for the simultaneous determination of vitamin D3 (CT and CHL) and K2 (MK-4 and MK-7) in dietary supplements. The obtained results were similar to those reported by Jehangir, which reported a recovery for commercial capsule formulations of 104% [15]. The proposed analytical method can be optimized and applied to other substances (also other vitamins), since methanol is one of the most versatile solvents due to its polarity; methanol can extract both polar and medium polar compounds.

## 3. Materials and Methods

### 3.1. Sample Collection

Six (*n =* 6) representative dietary supplements (DS, capsules) with individual or combined D3 and K2 vitamins in different concentration levels and two (*n =* 2) synthetic drugs (SD) were brought form the local pharmacies and specialized stores (Table 9).

### 3.2. Reagents, Solvents and Materials

All standard substances were HPLC grade: CT (calcitriol, vitamin D3, ≥97.9%, Ehrenstorfer, Augsburg, Germany), CHL (cholecalciferol, vitamin D3, ≥99.9%, Thermo Scientific, Waltham, MA, USA), MK-4 (vitamin K2, ≥99.2%, Chiron, Trondheim, Norway), and MK-7 (vitamin K2, ≥98.1%, Chiron, Trondheim, Norway). HPLC-grade solvents were supplied by Honeywell (Seelze, Germany). Syringe filters, labeled, Chromafil Xtra RC, 0.45 µm Macherey, Nagel, France. The deionized water used for all experiments was obtained from a Milli-Q Plus water purification system (Millipore, Bedford, MA, USA).

### 3.3. Preparation of Standard Solution

Individual and mixed standard stock solutions of 1 mg/mL of each analyte were prepared in methanol with different concentrations and stored in amber colored vials at −20 °C prior to use. The working standard solutions were prepared by diluting the stock solution in methanol.

### 3.4. Sample Preparation

The 0.1 g of well-blended dietary supplement samples were added in a centrifuge tube with 0.5 mL methanol and vortexed for 1 min at room temperature. Then, the extracts were centrifuged at 11,000 rpm for 1 min using a Hettich D-78532 centrifuge (Kirchlengern, Germany) and the supernatant was collected. The extraction was repeated from the residual mixture with 0.5 mL methanol. After each extraction, 1 mL supernatant solution was filtered through a 0.45 μm filter and collected in a 1.5 mL vial for UHPLC-DAD analysis.

### 3.5. UHPLC-DAD Analysis

The analysis was performed using an ultra-high-pressure liquid chromatograph Vanquish UHPLC system (Thermo Fisher Scientific, Germering, Germany) with a diode array detector (DAD HL, Dionex/Thermo Fisher Scientific, Germering, Germany) and separated on an Acclaim C30 150 × 46 µm, 5 µm (Themo Scientific, Sunnyvile, CA, USA) at 30 °C. The mobile phase was ultrapure water (A) and methanol (B). The isocratic elution was performed at 1 mL/min in a proportion of 98% B. The injection volume was 10 μL and the autosampler was set at 10 °C to ensure that no sample degradation occurred due to temperature. The DAD detector was set at 265 nm.

### 3.6. Method Validation

Method validation was accomplished by assessing the main performance parameters: linearity, working range, limit of detection (LoD), limit of quantification (LoQ), precision, accuracy, recovery, robustness, and conformity of chromatographic parameters.

#### 3.6.1. Linear Dynamic Range, Linearity, Limit of Detection (LOD), and Limit of Quantification (LOQ)

The linear dynamic range was selected within 10–200 μg/mL for vitamin D3 (CT and CHL) and 2–40 μg/mL for vitamin K2 (MK-4 and MK-7). The linear regression analysis was performed by plotting peak area (y) against the nominal concentrations (x), whereas a represented slope of the calibration curve and *b* indicated the intercept. The LOD and LOQ were estimated by the analysis of vitamins at low levels in the fortified samples; six replicates were used. The LOD was calculated as three times the standard deviation (SD) of the replicate fortified sample measurements and LOQ was ten times the standard deviation [16]. The repeatability of LOQ values was verified with sample matrix at the LOQ level.

#### 3.6.2. Recovery, Selectivity and Specificity

Because, to our knowledge, there are no certified reference materials for the analyzed vitamins in dietary supplements, the recovery was evaluated by analyzing three fortified samples of similar capsule content, with different D3 and K2 concentrations. The fortified samples were extracted and analyzed as specified in the method. The method accuracy was determined by comparison of the test result with the true value. The recovery efficiency, compared to the initial determination, must be between 80% and 120% [13].

#### 3.6.3. Precision

The method repeatability was determined by spiking six samples containing a similar capsule content with a known amount of each analyte (40 µg/g). The RSD of the repeatability should be lower than 2% [13]. In order to evaluate the intraday precision (repeatability) of the method, three lots of fortified samples containing similar capsule content and standard vitamin D3 and K2 at different concentration (10, 12, and 30 µg/mL for vitamin D3 (CT and CHL), and 5, 6, and 10 µg/mL for vitamin K2 (MK-4 and MK-7) were determined over five consecutive days.

#### 3.6.4. Robustness

The method robustness was evaluated by small, but cautious changes in the chromatographic parameters, namely: column temperature (±5 °C), flow rate (±0.1 mL/min), mobile phase ratio (±2%), and wavelength (±3 nm). The proposed changes should not lead to more than 10% changes in the measured concentrations or chromatographic parameters.

### 3.7. Analysis of Dietary Supplements

Six different dietary supplements and two synthetic drugs in commercial capsule form were extracted and analyzed using the proposed method. The obtained concentrations using the validated method were compared with those presented on the product labels.

## 4. Conclusions

In this study, an UHPLC method was fully validated according to European Pharmacopoeia guidelines for the simultaneous determination of vitamins D3 (calcitriol, cholecalciferol) and K2 (menaquinone-4 and menaquinone-7) in dietary supplements. All target analytes needed only one injection and could be separated and fast measured (<35 min). The validated UHPLC method displayed favorable linearity, good recoveries, intra-day and inter-day precisions, high selectivity and specificity, and low LODs and LOQs for all the target nutritional compounds (D3 and K2 vitamins). This study is of great value for the control analytical laboratories dealing with the simultaneous determination of vitamin D3 and K2 in dietary supplements.

## Figures and Tables

**Table 1:** Chromatographic parameters.

Parameter	CT	CHL	MK-4	MK-7
Retention time (min)	2.38	6.59	7.07	32.5
RSD of retention time (%)	0.87	0.06	0.26	0.21
Asymmetry factor	1.31	1.25	1.21	1.11
Resolution	6.17	8.91	2.02	-
Theoretical plates (N)	10,600	10,846	11,484	13,141

**Table 2:** Limit of detection (LOD) and limit of quantification (LOQ).

No crt.	CT	CHL	MK-4	MK-7
1	0.102	0.501	0.111	0.105
2	0.110	0.511	0.113	0.103
3	0.108	0.508	0.114	0.111
4	0.109	0.507	0.102	0.102
5	0.108	0.516	0.110	0.111
6	0.101	0.507	0.109	0.114
Average	0.106	0.508	0.110	0.108
SD	0.0038	0.0050	0.0043	0.0050
%RSD	3.60	0.98	3.88	4.61
n	6	6	6	6
LOD (µg/mL)	0.011	0.015	0.013	0.015
LOQ (µg/mL)	0.038	0.050	0.043	0.050

**Table 3:** Repeatability and recovery for the limit of quantification (LOQ).

No crt.	CT	CHL	MK-4	MK-7
1	0.05	0.06	0.04	0.07
2	0.05	0.07	0.04	0.04
3	0.05	0.05	0.03	0.06
4	0.05	0.05	0.04	0.06
5	0.04	0.07	0.05	0.05
6	0.05	0.04	0.05	0.06
SD	0.01	0.01	0.01	0.01
%RSD	13.1	17.8	15.3	16.8
Average (µg/mL)	0.05	0.06	0.04	0.06
Recovery (%)	115	111	104	112

**Table 4:** Method recovery.

Analyte	n	Fortified (µg/g)	Recovered (µg/g)	Recovered Mean (µg/g)	SD	RSD (%)	Recovery (%)
CT	3	12.5	10.5	10.6	0.28	2.60	85
			10.9				
			10.4				
CHL	3	12.6	10.4	10.3	0.35	3.37	81
			10.5				
			9.87				
MK-4	3	6.12	5.07	5.08	0.10	1.88	83
			5.18				
			4.99				
MK-7	3	6.08	4.97	4.97	0.11	2.11	82
			4.86				
			5.07				

**Table 5:** Method precision.

Sample	CT (µg/g)	CHL (µg/g)	MK-4 (µg/g)	MK-7 (µg/g)
S1	40.12	40.56	30.24	30.25
S2	41.03	39.02	30.25	31.06
S3	39.89	40.15	30.78	31.07
S4	40.12	40.23	29.97	30.48
S5	40.18	40.99	29.44	29.67
S6	41.07	41.08	30.74	30.47
Average	40.40	40.34	30.24	30.50
SD	0.51	0.75	0.50	0.53
%RSD	1.27	1.86	1.66	1.73

**Table 6:** Method reproducibility.

Sample	No crt.	CT (µg/mL)	CHL (µg/mL)	MK-4 (µg/mL)	MK-7 (µg/mL)
Lot 1	Day 1	10.0	10.2	5.31	5.22
	Day 2	10.7	10.4	5.20	5.13
	Day 3	10.2	10.6	4.94	4.91
	Day 4	10.5	10.2	5.31	5.31
	Day 5	10.3	10.0	5.17	5.39
	Average	10.4	10.3	5.19	5.20
	SD	0.26	0.24	0.15	0.18
	%RSD	2.53	2.33	2.93	3.57
Lot 2	Day 1	12.5	12.8	6.19	6.23
	Day 2	12.3	13.0	6.32	6.12
	Day 3	12.3	12.0	6.52	6.31
	Day 4	11.9	12.5	6.35	6.54
	Day 5	12.5	12.4	6.16	6.65
	Average	12.3	12.5	6.31	6.37
	SD	0.24	0.36	0.14	0.22
	%RSD	1.98	2.88	2.31	3.41
Lot 3	Day 1	30.2	29.2	10.3	10.6
	Day 2	30.5	30.7	10.9	10.4
	Day 3	31.0	31.5	10.2	10.9
	Day 4	30.9	30.9	10.2	10.3
	Day 5	30.1	31.0	10.3	9.94
	Average	30.5	30.6	10.4	10.4
	SD	0.40	0.89	0.27	0.34
	%RSD	1.31	2.90	2.62	3.25

**Table 7:** Method robustness.

Analyte	Chromatographic Parameters	Column Temperature (°C)		Flow Rate (mL/min)		Mobile Phase Ratio (%)		Wavelength (nm)	
		25	35	0.9	1.1	96 MeOH	100 MeOH	262	268
CT	Assay (%)	100	100	100	101	100	100	99	100
	Retention time (min)	2.46	2.14	2.42	2.27	2.45	2.14	2.40	2.40
	Asymmetry factor	1.41	1.39	1.40	1.32	1.41	1.29	1.30	1.31
	Resolution	5.23	5.98	5.12	6.08	4.98	5.66	6.02	6.15
CHL	Assay (%)	101	101	101	100	100	100	100	100
	Retention time (min)	6.84	6.46	6.68	6.48	6.90	6.32	6.59	6.58
	Asymmetry factor	1.54	1.28	1.53	1.31	1.61	1.24	1.26	1.25
	Resolution	7.65	8.02	7.16	8.99	7.68	8.64	8.89	8.87
MK-4	Assay (%)	100	100	100	100	100	101	101	100
	Retention time (min)	7.24	6.88	7.27	6.98	7.29	6.89	7.09	7.10
	Asymmetry factor	1.56	1.20	1.58	1.22	1.98	1.22	1.20	1.21
	Resolution	1.98	2.03	1.97	2.01	1.96	2.03	2.00	2.01
MK-7	Assay (%)	100	101	101	101	100	100	100	100
	Retention time (min)	32.7	32.4	32.8	32.5	32.8	32.3	32.5	32.5
	Asymmetry factor	1.34	1.09	1.42	1.11	1.39	1.09	1.10	1.10
	Resolution	100	100	100	101	100	100	100	100

**Table 8:** Assay results of vitamins D3 and K2 by HPLC in commercial dietary supplements.

Analyte	Sample	Concentration Found ± SD (µg/Capsule)	%RSD	Recovery (%)
**CT**	SD-CT1	0.256 ± 0.01	4.42	102
	SD-CT2	0.517 ± 0.01	2.05	103
**CHL**	DS-CHL@MK4	25.7 ± 0.57	2.20	103
	DS-CHL@MK7	6.21 ± 0.23	3.76	96
	DS-CHL	25.6 ± 0.35	1.38	103
	DS-CHL@MK7	125 ± 1.84	1.47	100
**MK-4**	DS-CHL@MK4	100 ± 1.54	1.54	100
	DS-MK4	52 ± 0.85	1.62	105
**MK-7**	DS-CHL@MK7A	51 ± 0.97	1.90	102
	DS-MK4@MK7	52 ± 0.63	1.21	104
	DS-CHL@MK7B	104 ± 1.80	1.74	104

**Table 9:** Dietary supplements and synthetic drugs analyzed (capsule form).

Sample	Concentration of Vitamins (µg/Capsule)			
	CT	CHL	MK-4	MK-7
DS-CHL@MK4	-	25	100	-
DS-MK4	-	-	100	-
DS-CHL@MK7A	-	6.25	-	50
DS-CHL	-	25	-	-
DS-MK4@MK7	-	-	50	50
DS-CHL@MK7B	-	125	-	100
SD-CT1	0.25	-	-	-
SD-CT2	0.5	-	-	-

## Data Availability

Not applicable.

## Sample Availability

Samples of the compounds are not available from the authors.
